# Unusual Presentation of Pyogenic Liver Abscess in a Healthy Young Pilot: A Case Report

**DOI:** 10.7759/cureus.79361

**Published:** 2025-02-20

**Authors:** Roukia Y Boucherabine, Abdellatif Hersh, Salli Aziz

**Affiliations:** 1 Internal Medicine, American Hospital Dubai, Dubai, ARE; 2 General Internal Medicine, American Hospital Dubai, Dubai, ARE

**Keywords:** liver abscess aspiration, pyogenic liver abscesses, sepsis treatment, shigella, streptococcus intermedius

## Abstract

Pyogenic liver abscess is a very rare, yet serious, condition, usually accompanied by different risk factors. In this article, we present the case of pyogenic liver abscess in an otherwise healthy male patient. What makes our patient's case unique is the fact that he does not have any past medical history and initially had very vague symptoms that were not very consistent with a diagnosis of a liver lesion, hence the delay in further testing and the conclusion of liver abscess diagnosis. Treatment includes draining of the abscesses, as well as a course of antibiotics. It is important to perform a comprehensive check-up after a liver abscess diagnosis, as it usually indicates an underlying disease. Early management is crucial to reduce the morbidity and mortality associated with the condition.

## Introduction

Pyogenic liver abscess (PLA) is a serious yet uncommon condition, with an occurrence of 2.3 per 100,000 annually and often arising as a complication of underlying infections or medical conditions [1]. It involves a localized collection of pus within the liver, typically caused by bacterial infiltration. While PLA is more commonly associated with risk factors such as diabetes, hepatobiliary diseases, or immunosuppression, it can occasionally present in otherwise healthy individuals, making early diagnosis challenging [2]. 

Treatment includes draining of the abscesses and a four- to six-week course of antibiotics. It is important to look for any underlying conditions when diagnosing a liver abscess, as it is usually a complication of another disease. A colonoscopy or tumor marker testing is warranted in these cases. Early management is highly encouraged to reduce morbidity and mortality associated with the condition [3].

This case report highlights the unique presentation of a young, healthy male pilot who developed PLA without any apparent predisposing factors. His initial symptoms were nonspecific, leading to a delayed diagnosis and a complex clinical course. Through a multidisciplinary approach involving imaging, microbiological analysis, and targeted antimicrobial therapy, the patient successfully recovered. This case underscores the importance of maintaining a high index of suspicion for PLA, even in patients without conventional risk factors, to ensure timely intervention and improved outcomes.

## Case presentation

This is the case of a 36-year-old male patient who works as a pilot with no past medical history, is a nonsmoker, and occasionally consumes alcohol. He presented to the emergency department with a one-day history of vomiting, diarrhea, persistent high-grade fever (up to 40°C), mild dry cough, and generalized fatigue after returning from a recent trip to Germany. He mentioned a 24-hour layover in Sweden during the trip. 

In the emergency department, investigations revealed left lower lobe infiltrates, and his blood tests revealed mild hyponatremia, hypokalemia, and elevated liver enzymes. On examination, the patient appeared tired with a high-grade fever (39.1°C) and was tachycardic at 128 bpm and borderline hypotensive at 97/61 mmHg. He also reported that he had dizziness and nausea. He denied any abdominal pain and did not have any tenderness on palpation.

A respiratory viral panel was ordered for him, which came back negative, as well as a urine analysis, which was also negative. Rapid *Streptococcus *test and urine *Legionella *antigen both came back negative. Prothrombin time (PT) and international normalized ratio (INR) were elevated, at 16s and 1.4, respectively. Alanine transaminase (ALT), aspartate aminotransferase (AST), and bilirubin were also elevated at 69 U/L, 51 U/L, and 33.8 µmol/L, respectively (Table 1).

**Table 1:** Laboratory results The results showed significantly elevated WBC count, neutrophils, CRP, procalcitonin, and liver enzymes and negative CA 19-9 marker WBC: white blood cell; INR: international normalized ratio; ALT: alanine aminotransferase; AST: aspartate aminotransferase; CRP: C-reactive protein; H: high; L: low

Parameters	Day 1	Day 2	Day 4	Day 8
WBC count (×10^9^/L)	22.8 (H)	21.4 (H)	11.3 (H)	13 (H)
Neutrophil absolute (×10^9^/L)	19.51 (H)	18.66 (H)	11.16 (H)	9.32 (H)
Hemoglobin (g/L)	165.0 (H)	151.0 (H)	-	-
Neutrophil %	85.7 (H)	87.0 (H)	-	-
Prothrombin time (s)	16.0 (H)	19.2 (H)	-	-
INR	1.4 (H)	1.6 (H)	-	-
Activated partial thromboplastin time (s)	28.7 (H)	31.4 (H)	-	-
Albumin (g/L)	32 (L)	29 (L)	-	-
Alkaline phosphatase (U/L)	79	110 (H)	-	-
ALT (U/L)	69 (H)	103 (H)	67 (H)	-
AST (U/L)	51 (H)	60 (H)	-	-
Bilirubin direct (µmol/L)	33.8 (H)	6.4 (H)	-	-
Procalcitonin (ng/mL)	2.90 (H)	9.22 (H)	5.35 (H)	0.34
CRP (mg/L)	208 (H)	265 (H)	296 (H)	131 (H)
CA 19-9 (U/mL)	14.8	-	-	-

The patient was started on ceftriaxone and azithromycin, but due to poor response, he was switched to levofloxacin. He continued to have high-grade fever and persistent cough. The diarrhea resolved but he still had poor oral intake. Malaria, dengue, *Salmonella*, hepatitis panel, *Cytomegalovirus* (CMV), and Epstein-Barr virus (EBV) screening were all done to exclude other underlying causes of infection (Table 2). 

**Table 2:** Infectious diseases panel *Shigella*/EIEC PCR came back positive indicating an infection EIEC: enteroinvasive *E. coli*; PCR: polymerase chain reaction; CMV: *Cytomegalovirus *

Infectious panel	Value
*Shigella*/EIEC PCR	Positive
Hepatitis A IgM	Negative
Hepatitis B IgM	Negative
Hepatitis C IgM	Negative
Entamoeba histolytica	Negative
CMV DNA	Negative
Widal test	Negative
Dengue PCR	Negative

The rest of the results came back. Tumor markers were negative (Table 1). The hepatitis panel, CMV, and EBV were negative. Dengue and Widal tests were also negative (Table 2). The malaria blood film was also negative. Blood culture, which was also collected initially, is negative.

**Figure 1:**
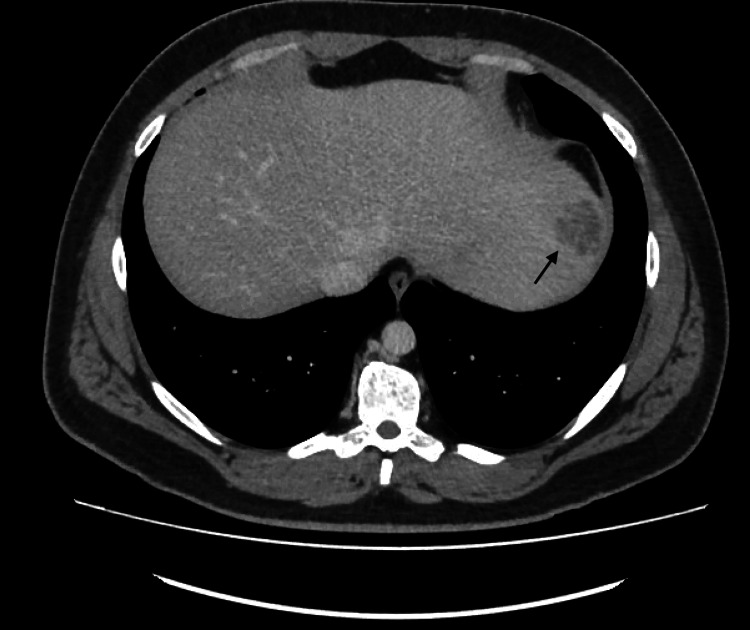
CT abdomen CT abdomen showing a lesion on the upper left lobe of the liver (as shown by the arrow)

**Figure 2:**
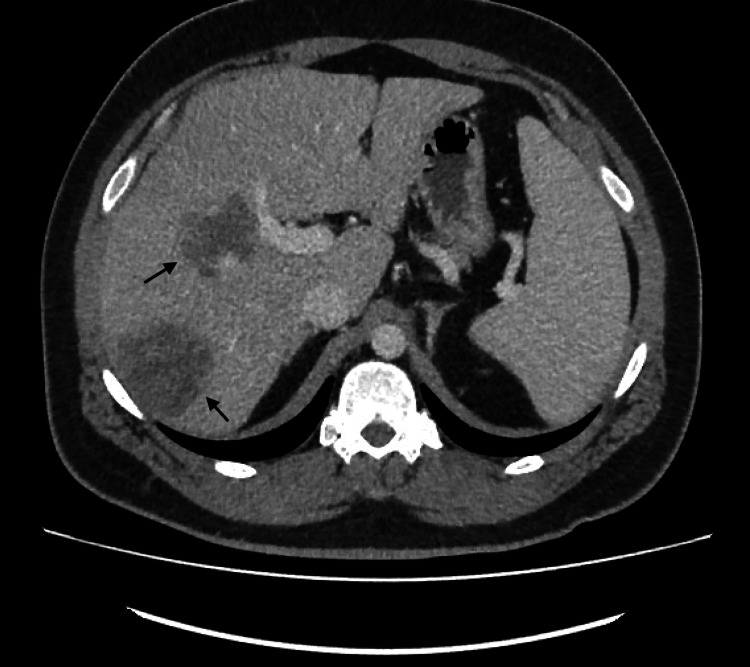
CT abdomen CT abdomen showing lesions in the middle (arrow above) and inferior right lobe (arrow below) of the liver

**Figure 3:**
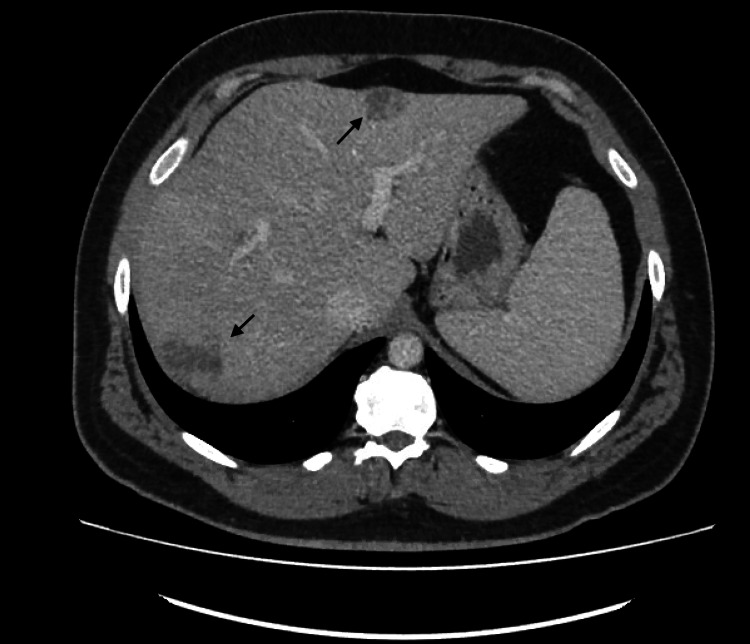
CT abdomen CT abdomen showing lesions on the anterior (arrow above) and inferior (arrow below) aspects of the right lobe of the liver

CT abdomen/pelvis (Figures 1-3) was ordered to exclude intra-abdominal sources of infection. Meanwhile, the patient was restarted on ceftriaxone to cover possible *Salmonella* infection. 

The CT abdomen showed liver masses, the largest measuring 5×5 cm suggestive of either metastasis or abscess collections, as they appeared as hypodense lesions without any rim enhancement, internal septations, or fluid levels. An interventional radiologist was consulted and scheduled the patient for drainage for possible abscess collection.

A gastroenterologist was also consulted and advised to send for tumor markers such as carcinoembryonic antigen (CEA) and CA 19-9, stool gastrointestinal polymerase chain reaction (PCR), stool culture, and occult blood. He advised starting the patient on metronidazole and continuing the ceftriaxone as his C-reactive protein (CRP) was significantly elevated at 268 and procalcitonin reached 9.22 (Table 1).

The surgery team was consulted and did not advise any surgical intervention. Stool PCR came back positive for *Shigella*. The interventional radiologist performed the ultrasound-guided percutaneous drainage, with two drainable pockets, each measuring 15 mm and 35 mm, and sent the fluid for culture and sensitivity. During this time, the patient started to respond slowly to the current regimen of antibiotics, evident in the drop in the inflammatory marker numbers and spacing out of the fever intervals.

The fluid culture showed heavy growth of *Streptococcus intermedius*. The patient's antibiotics were then changed to meropenem and continued with metronidazole. He was continuously feeling better and reported occasional episodes of diarrhea, but no abdominal pain. His WBC counts were improving and so were his liver enzymes and CRP and procalcitonin levels. 

The culture sensitivity showed that the organism was susceptible to cefotaxime, ceftriaxone, and penicillin, so the patient was discharged on 2 g ceftriaxone IV daily for approximately three weeks and instructed to follow up in one week with the infectious diseases consultant to potentially change the IV antibiotics to an oral one to complete a total of six-week antibiotic course. A repeat ultrasound in three weeks is needed for further monitoring.

## Discussion

PLA is an uncommon but dangerous illness, usually brought on by bacterial infections. It is marked by a localized buildup of pus inside the liver parenchyma. PLA in an otherwise healthy adult poses a special clinical challenge, even though the majority of instances happen in people with predisposing conditions including diabetes, hepatobiliary disorders, or immunosuppression [4]. 

Different cases have been discussed in the literature; however, most of them have always had a possible underlying cause, such as the case of an 18-year-old, previously healthy male patient, who presented with fever, abdominal pain, and emesis, but had a preceding history of upper respiratory tract infection a week before the presentation; thus, it could've been a potential cause of bacteremia leading to the abscesses [5]. Another case discussed a 35-year-old male patient, with no past medical history, who presented with a six-week history of cough, fever, malaise, and anorexia. On further examination and investigations, he was found to have absent breath sounds on the right lower lung and diffuse opacification on chest X-ray, consistent with the diagnosis of pneumonia with parapneumonic effusion. Therefore, he also had an underlying likely cause of infection in the blood, which could have led to the formation of liver abscesses [6].

PLA commonly arises from ascending infections via the biliary tract, hematogenous spread, or direct extension from adjacent structures. The most frequently isolated organisms are *Escherichia coli* and *Klebsiella pneumoniae*, though polymicrobial infections are not uncommon [7]. In healthy individuals, cryptogenic abscesses may occur, where no clear source of infection is identified [2].

The symptoms, which include fever, malaise, stomach pain (particularly in the right upper quadrant), and sometimes jaundice, are frequently nonspecific. Because it might overlap with other intra-abdominal disorders, diagnosis can be difficult. Finding the abscess depends heavily on imaging, especially contrast-enhanced CT or ultrasonography [8]. Microbiological analysis using blood cultures or aspirates from percutaneous drainage is necessary for a conclusive diagnosis [3]. In the case discussed above, our patient had done an X-ray which showed a suspicious consolidation-like lesion, which warranted the CT abdomen/chest and finally led to the incidental diagnosis of the liver lesions on the view of the upper liver segments.

PLAs can lead to severe complications and pose significant health risks if not promptly diagnosed and treated. The infection can disseminate into the bloodstream leading to systemic inflammatory response syndrome, sepsis, and potentially septic shock [9]. Pleural effusion is another complication, which can happen if the inflammation extends to the diaphragm and pleura, resulting in fluid accumulation in the pleural space, in approximately 34.5% of PLA cases [9]. This condition is associated with many other dangerous complications which is why it is important to treat it as soon as possible. The mortality rate associated with this condition has decreased recently due to advancements in diagnostic and therapeutic approaches, but it still ranges from 11% to 31% depending on different underlying factors [4].

## Conclusions

The management of PLA is heavily dependent on a multidisciplinary approach, as it is essential to involve internists, gastroenterologists, interventional radiologists, and infectious diseases specialists to tailor the best possible plan. The treatment involves a combination of drainage of the abscess, either percutaneously or surgically, and tailored antimicrobial therapy according to the culture results. Prompt diagnosis and management are crucial to prevent complications such as rupture, sepsis, or multiorgan failure.

In healthy adults, PLA may indicate a previously undetected underlying condition, such as an occult malignancy or gastrointestinal pathology. Therefore, a comprehensive workup, including a colonoscopy, may be warranted. Despite its rarity, timely recognition and management of PLA in healthy individuals are critical to ameliorating outcomes and reducing morbidity.
